# Influence of Dexmedetomidine on Post-operative Atrial Fibrillation After Cardiac Surgery: A Meta-Analysis of Randomized Controlled Trials

**DOI:** 10.3389/fcvm.2021.721264

**Published:** 2021-11-25

**Authors:** Sheng Peng, Juan Wang, Hui Yu, Ge Cao, Peirong Liu

**Affiliations:** ^1^Department of Anesthesiology, Seventh People's Hospital of Shanghai University of Traditional Chinese Medicine, Shanghai, China; ^2^Department of Cardiovascular Surgery, Shanxi Fenyang Hospital, Fenyang, China

**Keywords:** dexmedetomidine, post-operative atrial fibrillation (POAF), perioperative, cardiac surgery, meta-analysis

## Abstract

**Background:** Previous clinical studies and meta-analysis evaluating the influence of dexmedetomidine on postoperative atrial fibrillation showed inconsistent results. We performed an updated meta-analysis to evaluate the influence of dexmedetomidine on incidence of postoperative atrial fibrillation after cardiac surgery.

**Methods:** Randomized controlled trials that evaluated the potential influence of dexmedetomidine on the incidence of atrial fibrillation after cardiac surgery were obtained by search of PubMed, Embase, and Cochrane's Library databases from inception to April 12, 2021. A random-effects model incorporating the potential publication bias was used to pool the results. Influences of patient or study characteristics on the efficacy of dexmedetomidine on atrial fibrillation after cardiac surgery were evaluated by meta-regression and subgroup analyses.

**Results:** Fifteen studies with 2,733 patients were included. Pooled results showed that dexmedetomidine significantly reduced the incidence of atrial fibrillation compared to control (OR: 0.72, 95% CI: 0.55–0.94, *p* = 0.02) with mild heterogeneity (*I*^2^ = 26%). Subgroup analysis showed that dexmedetomidine significantly reduced the incidence of atrial fibrillation in studies from Asian countries (OR: 0.41, 95% CI: 0.26–0.66, *p* < 0.001), but not in those from non-Asian countries (OR: 0.89, 95% CI: 0.71–1.10, *p* = 0.27; *p* for subgroup difference = 0.004). Meta-regression analysis showed that the mean age and proportion of male patients may modify the influence of dexmedetomidine on POAF (coefficient = 0.028 and 0.021, respectively, both *p* < 0.05). Subgroup analysis further showed that Dex was associated with reduced risk of atrial fibrillation after cardiac surgery in studies with younger patients (mean age ≤ 61 years, OR = 0.44, 95% CI: 0.28–0.69, *p* = 0.004) and smaller proportion of males (≤74%, OR = 0.55, 95% CI: 0.36–0.83, *p* = 0.005), but not in studies with older patients or larger proportion of males (*p* for subgroup difference = 0.02 and 0.04).

**Conclusions:** Current evidence supports that perioperative administration of dexmedetomidine may reduce the risk of incidental atrial fibrillation after cardiac surgery, particularly in Asians.

## Introduction

Post-operative atrial fibrillation (POAF) is a common complication, which affects up to 35% of patients after cardiac surgery (1, 2). Clinically, POAF has been related with a higher incidence of neurocognitive dysfunction, prolonged hospital stay, and increased risk of all-cause mortality in these patients (3). Current risk factors for POAF after cardiac surgery include aging, diabetes mellitus, and impaired cardiac function etc. (4). However, no definite strategy has been confirmed as an effective preventative method for POAF (5, 6). Dexmedetomidine (Dex) is well-applied perioperative medication for patients that received cardiac surgery (7). Pharmacologically, Dex is a highly selective α2-adrenoreceptor agonist which exerts various clinical efficacies during perioperative periods, such as sedation, analgesia, anti-anxiety, and diuresis (8, 9). Besides, use of Dex is suggested to reduce POAF after cardiac surgeries in some previous randomized controlled trials (RCTs) (10–12), although other RCTs generally did not support a preventative efficacy of Dex for POAF (13–24). A few meta-analyses have been performed to evaluate the influence of Dex on POAF after cardiac surgery. Early meta-analyses including RCTs before 2017 consistently showed that Dex was not associated with reduced incidence of POAF after cardiac surgeries (25–29), while a recent meta-analysis including RCTs up to 2018 showed that Dex was effective in preventing POAF after cardiac surgeries (30). However, results of this meta-analysis are confounded by including two RCTs with overlapped patients (10, 31), which both showed a significant preventative efficacy of Dex on POAF. Besides, three RCTs have been published since the last meta-analysis (12, 23, 24), including a large-scale RCT which suggested a lack of preventative efficacy of Dex on POAF (24). Accordingly, we performed an updated meta-analysis to systematically evaluate the possible influence of Dex on POAF after cardiac surgery. Multiple predefined subgroup analyses were also performed to explore the potential influences of patient or study characteristics on the outcome.

## Methods

We followed the instructions of the PRISMA (Preferred Reporting Items for Systematic Reviews and Meta-Analyses) statement (32) and the Cochrane Handbook guidelines (33) during the designing, performing, and reporting of the meta-analysis.

### Search Strategy

PubMed, Embase, and the Cochrane's Library (Cochrane Center Register of Controlled Trials) databases were searched for relevant studies with a combined strategy of: (1) “dexmedetomidine”; (2) “heart” OR “cardiac surgery” OR “coronary artery bypass graft” OR CABG OR “atrial fibrillation” OR arrhythmia OR “cardiac”; and (3) “random” OR “randomized” OR “randomised” OR “randomly.” Only clinical studies were considered. The references of related reviews and original articles were also searched as complementation. The latest database search was performed on April 12, 2021.

### Study Selection

Inclusion criteria were: (1) peer-reviewed articles in English; (2) designed as parallel-group RCTs; (3) included adult patients scheduled for open heart surgery who were randomly allocated to a perioperative administration of Dex or a non-Dex control group; and (4) reported the incidence of POAF during the index hospitalization. For studies with overlapped patients, the one with the largest sample size was included (33). Reviews, studies including children or neonates, preclinical studies, observational studies, and repeated reports were excluded.

### Data Extraction and Quality Assessment

Study search, data extraction, and quality evaluation were conducted by two independent authors. If disagreement occurred, it was resolved by consensus between the two authors. We extracted data regarding study information (first author, publication year, and study country), study design (blind or open-label), patient information [number of participants, mean age, gender, body weight or body mass index (BMI), and surgery type], details of Dex administration (regimen, timing and duration), and components of controls. Quality evaluation was achieved using the Cochrane's Risk of Bias Tool (33) according to the following aspects: (1) random sequence generation; (2) allocation concealment; (3) blinding of participants and personnel; (4) blinding of outcome assessors; (5) incomplete outcome data; (6) selective outcome reporting; and (7) other potential bias.

### Statistical Analysis

Incidence of POAF in each arm was evaluated via odds ratio (OR) and its 95% confidence intervals (CIs). We used the Cochrane's Q test to detect the heterogeneity, and significant heterogeneity was suggested if *P* < 0.10 (34). The *I*^2^ statistic was also calculated, and an *I*^2^ > 50% reflected significant heterogeneity. Pooled analyses were calculated using a random-effect model because this method could incorporate the influence of potential heterogeneity and retrieves a more generalized result (33). Sensitivity analysis by excluding one study at a time was used to determine the robustness of the finding. Subgroup analyses were performed to determine the potential influences of study characteristics on the results of meta-analysis, including country of the study, type of surgery, mean age of the patients, proportions of the males, with or without loading dose of Dex, timing of Dex administration, regimen of controls, or score of study quality. Meta-regression analyses were also performed to evaluate the potential influences of mean age of the patients and proportions of the males on the effect of Dex on POAF. Medians of continuous variables were used as cut-off values for defining of subgroups. Publication bias was evaluated by visual inspection of funnel plots, and the Egger's regression asymmetry test (35). *P*-values <0.05 were considered statistically significant. The RevMan (Version 5.1; Cochrane, Oxford, UK) and Stata software (Version 12.0; Stata, College Station, TX) were applied for statistical analyses.

## Results

### Search Results

**Figure 1:**
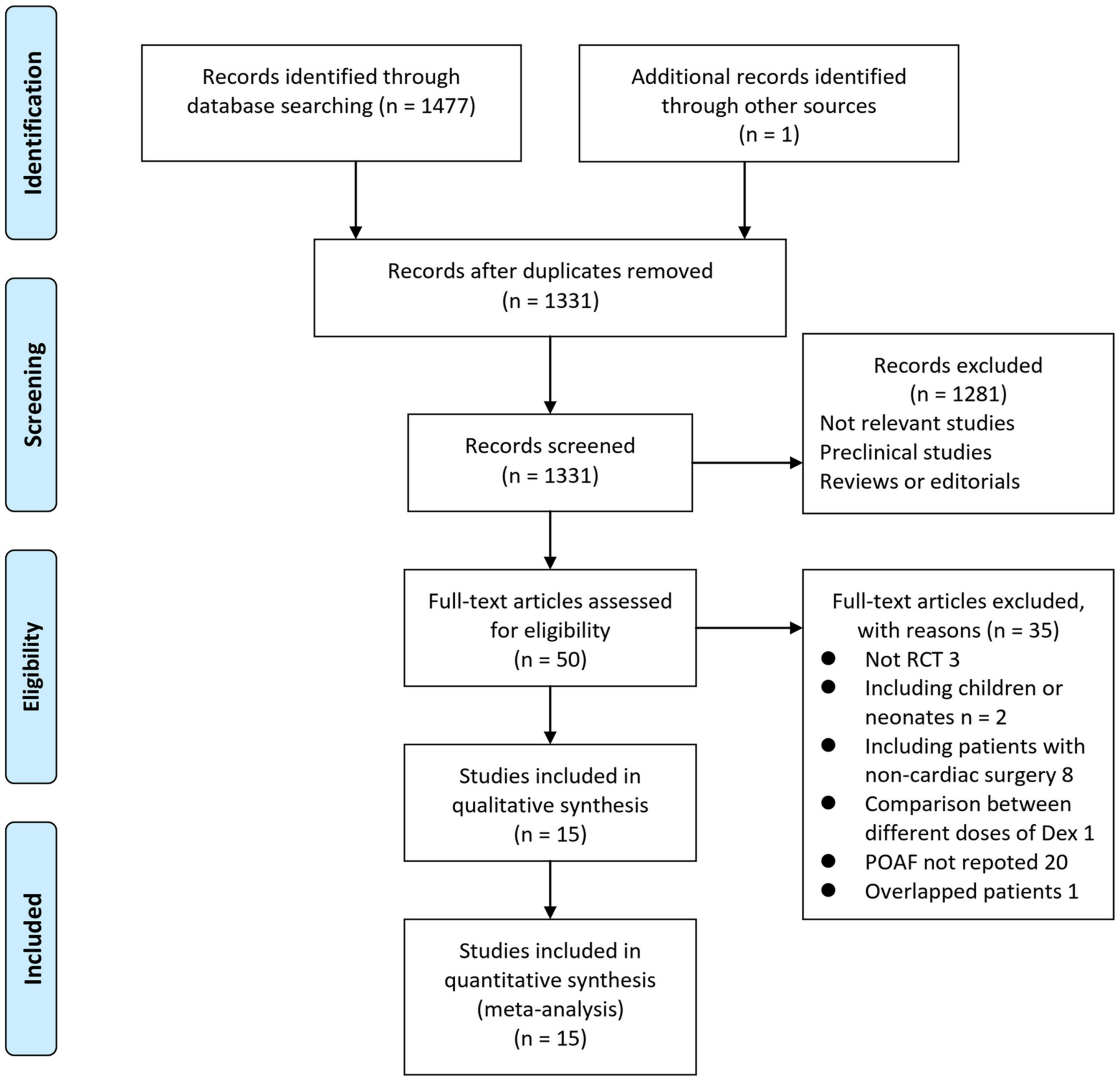
Flowchart of literature search.

In summary, 1,478 articles were obtained through the database search. After exclusion of duplicate studies, 1,331 articles were screened. Among them, 1,281 articles were subsequently excluded based on titles and abstracts primarily because these studies were irrelevant. Among the 50 potentially relevant articles, 35 were further excluded via full-text review based on reasons listed in Figure 1. Finally, 15 RCTs (10–24) were included into the meta-analysis.

### Study Characteristics

Table 1 shows the characteristics of the included studies. Overall, 15 RCTs (10–24) with 2,733 patients who received cardiac surgery – 1,388 patients in the Dex group and 1,345 patients in the control group - were included. These studies were published between 1997 and 2020. All of the studies included patients that underwent open-heart cardiac surgery. Among them, ten RCTs included patients who underwent elective coronary artery bypass grafting (CABG) (11–21), while the other five included patients who underwent mixed type of open-heart surgeries, including CABG, valvular surgeries, and aortic surgeries (10, 16, 22–24). The sample sizes of the included RCTs varied between 64 and 794. The mean ages of the patients varied between 52 and 75 years, with proportions of male ranging from 32 to 90%. Mean body weight of the patients was reported in five studies (11, 13–15, 22), while BMI was reported in another five studies (10, 16, 17, 21, 24). Dex was administered only during the procedure of surgery in two studies (13, 23), only during the stay in Intensive Care Unit (ICU) in nine studies (10, 12, 15–17, 19–22), and during the surgery and ICU in four studies (11, 14, 18, 24). A loading dose and subsequent continuously intravenous administration of Dex was applied five studies (13–15, 19, 22), and in the remaining ten studies, no leading dose of Dex was applied (10–12, 16–18, 20, 21, 23, 24). As for the controls, in six studies, Dex was compared to placebo (11, 13, 17, 18, 20, 24), while in the others, comparisons were between Dex and active controls including propofol (10, 12, 14, 15, 21–23), morphine (16), and remifentanil (19).

**Table 1:** Characteristics of the included clinical trials.

**Study**	**Country**	**Design**	**Surgery type**	**Sample size**	**Mean age**	**Male**	**Mean BW**	**Mean BMI**	**Dex regimens**	**Loading dose of Dex**	**Timing and duration of Dex**	**Control**
					**years**	**%**	**Kg**	**Kg/m^**2**^**				
Jalonen et al. (13)	Finland	R, DB, PC	CABG	80	55.4	83.8	80.4	NR	50 ng/kg/min for 30 min before induction of anesthesia, and 7 ng/kg/min until the end of surgery	Yes	During surgery	Saline
Herr et al. (14)	USA and Canada	R, OL	CABG	295	62.2	89.8	84.5	NR	At sternal closure, 1.0 μg/kg over 20 min and then 0.2–0.7 μg/kg/h to maintain during assisted ventilation	Yes	During surgery and ICU	Propofol
Corbett et al. (15)	USA	R	CABG	89	62.9	82	88.8	NR	1 ug/kg over 15 mins, followed by a 0.4 ug/kg/h initiated after bypass and during assisted ventilation	Yes	After surgery and in ICU	Propofol
Shehabi et al. (16)	Australia	R, DB	Mixed	299	71.3	75.3	NR	27.5	0.1–0.7 μg/kg/h	No	After surgery and in ICU	Morphine
Ren et al. (18)	China	R, SB, PC	CABG	162	59	32.5	NR	NR	0.2–0.5 μg/kg/h following the first vascular anastomosis grafting and last until transferred to ICU for 12 h	No	During surgery and ICU	Saline
Göksedef et al. (17)	Turkey	R, DB, PC	CABG	86	60.7	73.3	NR	26.6	0.5 μg/kg/h	No	After surgery and in ICU	Placebo (NR)
Park et al. (19)	Korea	R	CABG	142	52.7	56	NR	NR	loading dose: 0.5 μg/kg, and 0.2–0.8 μg/kg/h continuously	Yes	After surgery and in ICU	Remifentanil
Balkanay et al. (20)	Turkey	R, DB, PC	CABG	88	60.5	74.1	NR	NR	0.04–0.5 μg/kg/h	No	After surgery and in ICU	Placebo (NR)
Karaman et al. (21)	Turkey	R, OL	CABG	64	63.1	84.4	NR	27	0.2–1.0 μg/kg/h	No	After surgery and in ICU	Propofol
Djaiani et al. (22)	Canada	R, DB	Mixed	183	72.5	75.4	80.9	NR	0.4 μg/kg bolus followed by 0.2–0.7 μg/kg/h	Yes	After surgery and in ICU	Propofol
Liu et al. (31)	China	R, OL	Mixed	88	54.7	60.2	NR	22.1	0.2–1.5 μg/kg/h	No	After surgery and in ICU	Propofol
Soltani et al. (11)	Iran	R, DB, PC	CABG	76	59.8	40.8	72.8	NR	0.5 μg/kg/h	No	During surgery and ICU	Saline
Shi et al. (23)	China	R, DB	Mixed	164	74.5	72.6	NR	NR	0.4–0.6 μg/kg/h	No	During surgery	Propofol
Zi et al. (12)	China	R, DB	CABG	123	65.2	67.5	NR	NR	0.2–1.0 μg/kg/h	No	After surgery and in ICU	Propofol
Turan et al. (24)	USA	R, DB, PC	Mixed	794	62.5	69.6	NR	29	0.1–1.0 μg/kg/h	No	During surgery and ICU	Saline

*Dex, dexmedetomidine; R, randomized; DB, double-blind; SB, single-blind; OL, open-label; PC, placebo-controlled; CABG, coronary artery bypass grafting; ICU, intensive care unit; BW, body weight; BMI, body mass index; NR, not reported*.

### Data Quality

Table 2 shows the details of study quality evaluation. Nine of the included studies were double-blind (11–13, 16, 17, 20, 22–24), while the rest were single-blind (18) or open-label (10, 14, 15, 19, 21). Methods of random sequence generation were reported in seven studies (10, 12, 15, 16, 22–24), and information of allocation concealment was reported in five studies (11, 14, 21, 22, 24). The quality of the included studies varied, with a quality score from 3 to 7.

**Table 2:** Details of study quality evaluation via the Cochrane's Risk of Bias Tool.

**Study**	**Random sequence generation**	**Allocation concealment**	**Blinding of participants**	**Blinding of outcome assessment**	**Incomplete outcome data addressed**	**Selective reporting**	**Other sources of bias**	**Total**
Jalonen et al. (13)	Unclear	Unclear	Low	Low	Low	Low	Low	5
Herr et al. (14)	Unclear	Low	High	Unclear	Low	Low	Low	4
Corbett et al. (15)	Low	Unclear	Unclear	Unclear	Low	Low	Low	4
Shehabi et al. (16)	Low	Unclear	Low	Low	Low	Low	Low	6
Ren et al. (18)	Unclear	Unclear	Low	Unclear	Low	Low	Low	4
Göksedef et al. (17)	Unclear	Unclear	Low	Low	Low	Low	Low	5
Park et al. (19)	Unclear	Unclear	Unclear	Unclear	Low	Low	Low	3
Balkanay et al. (20)	Unclear	Unclear	Low	Low	Low	Low	Low	5
Karaman et al. (21)	Unclear	Low	High	High	Low	Low	Low	4
Djaiani et al. (22)	Low	Low	Low	Low	Low	Low	Low	7
Liu et al. (31)	Low	Unclear	High	High	Low	Low	Low	4
Soltani et al. (11)	Unclear	Low	Low	Low	Low	Low	Low	6
Shi et al. (23)	Low	Unclear	Low	Low	Low	Low	Low	6
Zi et al. (12)	Low	Unclear	Low	Low	Low	Low	Low	6
Turan et al. (24)	Low	Low	Low	Low	Low	Low	Low	7

### Meta-Analysis Results

**Figure 2:**
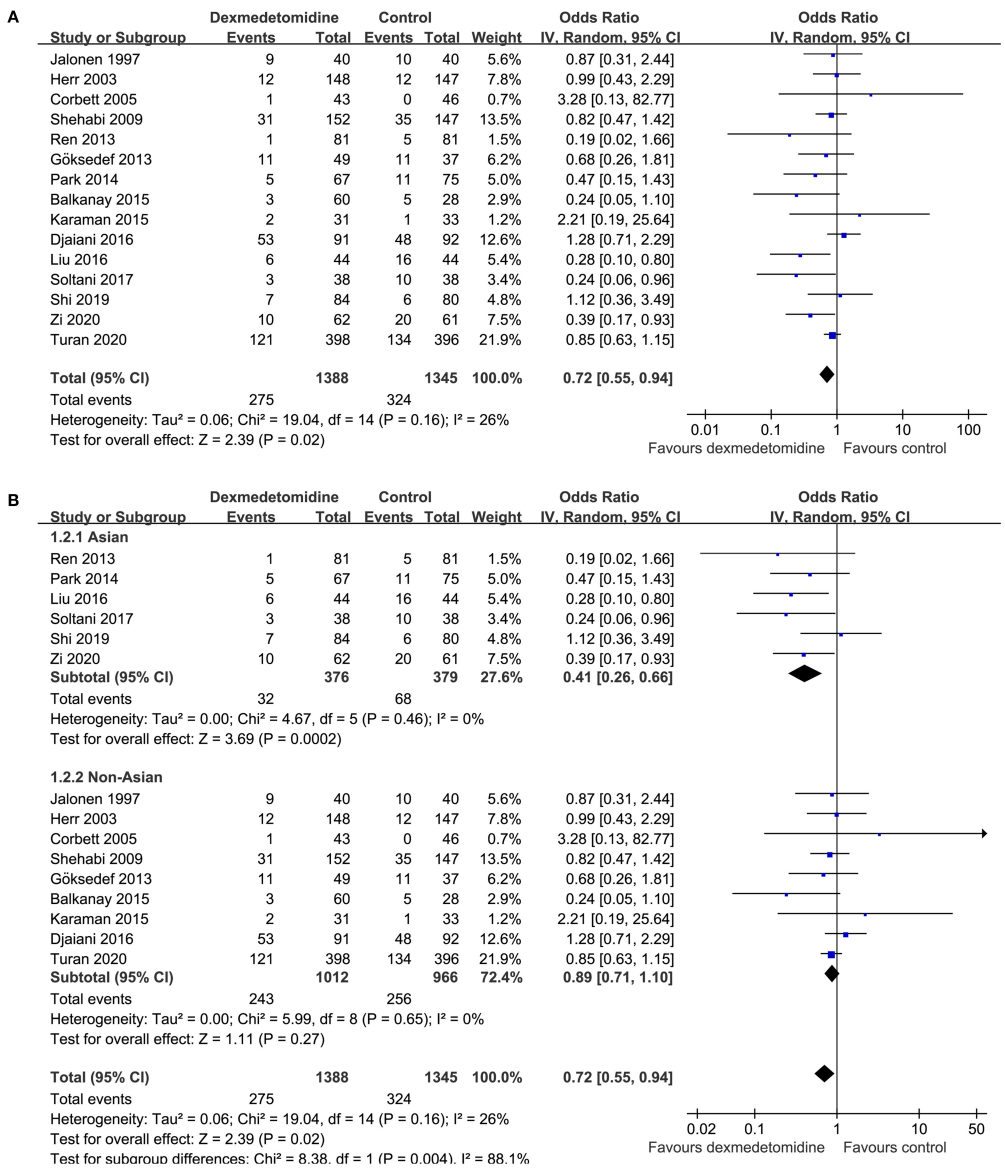
Forest plots for the meta-analysis comparing the influences of dexmedetomidine and controls on the incidence of POAF after cardiac surgery; **(A)** overall meta-analysis; and **(B)** subgroup analysis according to the origin of the studies.

Mild heterogeneity was detected among the included RCTs (p for Cochrane's Q test = 0.16, *I*^2^ = 26%). Pooled results with a random-effect model showed that Dex significantly reduced the incidence of POAF after cardiac surgery compared to control (OR: 0.72, 95% CI: 0.55–0.94, *p* = 0.02; Figure 2A). Sensitivity analysis by excluding one study at a time did not significantly affect the results (OR: 0.72–0.80, *p* all < 0.05). Subgroup analysis according to the origin of the study showed that Dex significantly reduced the incidence of atrial fibrillation in studies from Asian countries (OR: 0.41, 95% CI: 0.26–0.66, *p* < 0.001), but not in those from non-Asian countries (OR: 0.89, 95% CI: 0.71–1.10, *p* = 0.27; *p* for subgroup difference = 0.004; Figure 2B), which fully resolved the heterogeneity (*I*^2^ = 0% for both subgroups). Results of meta-regression analysis showed that the mean age and proportion of male patients may modify the influence of dexmedetomidine on POAF (coefficient = 0.028 and 0.021, respectively, both *p* < 0.05; Table 3). Further subgroup analyses showed that that Dex was associated with reduced POAF in studies with younger patients (mean age ≤ 61 years, OR: 0.44, 95% CI: 0.28–0.69, *p* = 0.004) and smaller proportion of males (≤ 74%, OR: 0.55, 95% CI: 0.36–0.83, *p* = 0.005; Table 4), but not in studies with older patients or larger proportion of males (*p* for subgroup difference = 0.02 and 0.04, respectively). The results were not significantly affected by difference in study characteristics such as type of surgery, with or without loading dose of Dex, timing of Dex administration, regimen of controls, or score of study quality (Table 4; *p* for subgroup analyses all > 0.05).

**Table 3:** Results of univariate meta-regression analysis.

**Study characteristics**	**OR for the incidence of POAF after cardiac surgery**		
	**Coefficient**	**95% CI**	***p***
Mean age (years)	0.028	0.004–0.052	0.027
Male (%)	0.021	0.003–0.039	0.015

*OR, odds ratio; POAF, post-operative atrial fibrillation; CI, confidence interval*.

**Table 4:** Results of subgroup analyses.

**Study characteristics**	**Datasets number**	**OR (95% CI)**	***I* ^**2**^**	***P* for subgroup effect**	***P* for subgroup difference**
**Country of study**
Asian	6	0.41 (0.26, 0.66)	0%	0.002	
Non-Asian	9	0.89 (0.71, 1.10)	0%	0.27	0.004
**Surgery type**
CABG	10	0.57 (0.39, 0.84)	3%	0.005	
Mixed	5	0.85 (0.60, 1.20)	38%	0.17	0.36
**Mean age (years)**
≤ 61	7	0.44 (0.28, 0.69)	0%	0.004	
>61	8	0.88 (0.71, 1.10)	0%	0.26	0.02
**Male (%)**
≤ 74	8	0.55 (0.36, 0.83)	38%	0.005	
>74	7	0.95 (0.69, 1.32)	0%	0.77	0.04
**Loading dose of Dex**
Yes	5	1.01 (0.68, 1.51)	0%	0.95	
No	10	0.60 (0.42, 0.86)	35%	0.006	0.09
**Dex duration**
Only in surgery	2	0.98 (0.45, 2.09)	0%	0.95	
Only in ICU	9	0.64 (0.42, 0.99)	39%	0.04	
In surgery and ICU	4	0.68 (0.38, 1.21)	40%	0.19	0.65
**Controls**
Placebo	6	0.62 (0.39, 0.99)	30%	0.04	
Propofol	7	0.80 (0.46, 1.37)	45%	0.41	
Others	2	0.74 (0.45, 1.20)	0%	0.22	0.77
**Quality score**
3–5	9	0.60 (0.39, 0.93)	9%	0.02	
6–7	6	0.80 (0.56, 1.13)	40%	0.20	0.32

*OR, odds ratio; CI, confidence interval; Dex, dexmedetomidine; CABG, coronary artery bypass grafting; ICU, intensive care unit; NR, not reported*.

### Publication Bias

**Figure 3:**
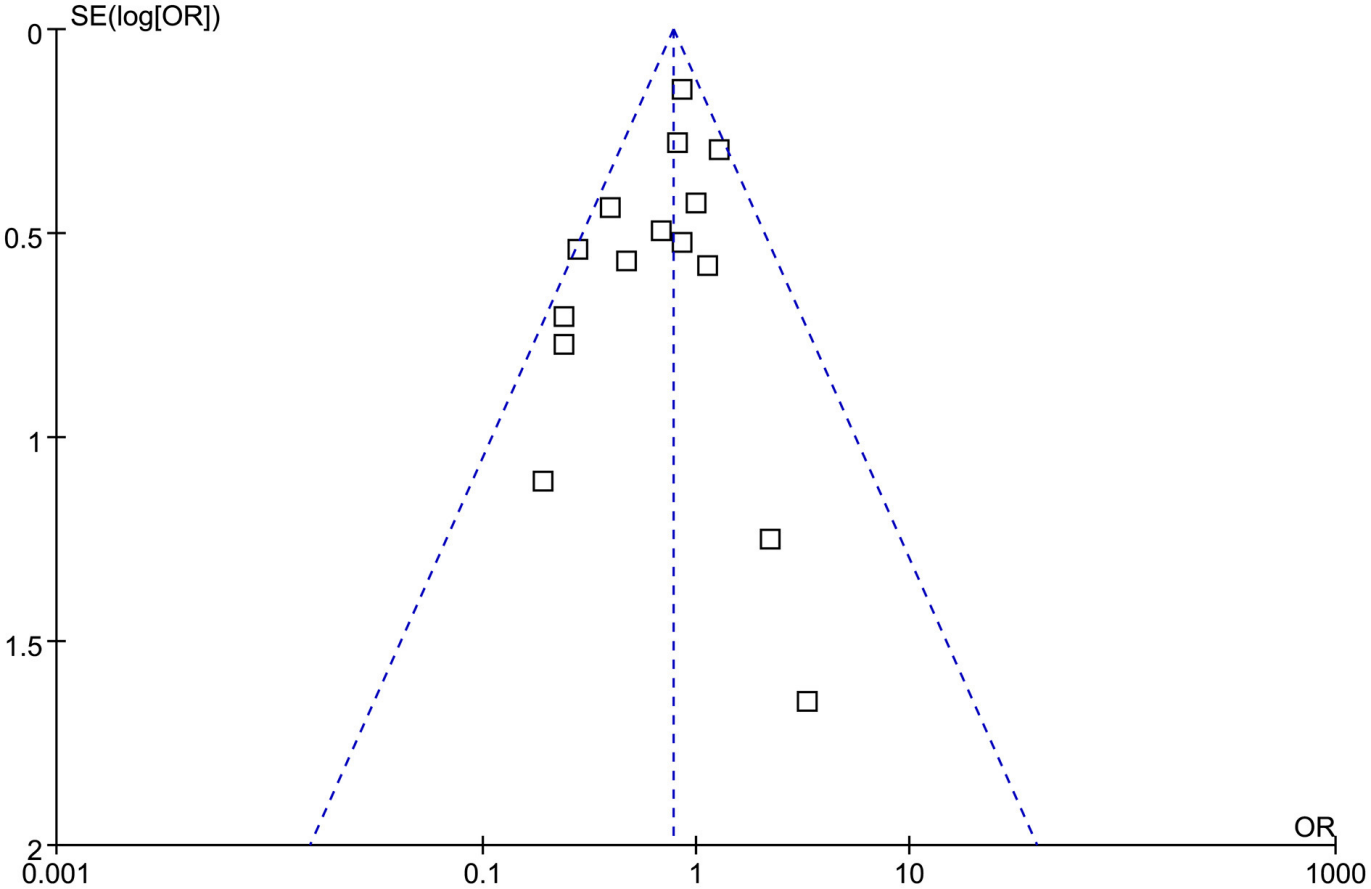
Funnel plots for the meta-analysis comparing the influences of dexmedetomidine and controls on the incidence of POAF after cardiac surgery.

The funnel plots were symmetrical, suggesting low-risk of publication bias (Figure 3). Egger's regression tests showed similar results (*p* = 0.301).

## Discussion

In this updated meta-analysis of RCTs, we found that perioperative use of Dex is associated with 28% reduction of POAF after cardiac surgeries. The results seem to be stable since sensitivity analysis by excluding one study a time showed consistent results. Besides, subgroup analysis showed that Dex was associated with reduced POAF in Asian studies, but not in non-Asian studies. Moreover, both meta-regression and subgroup analyses showed that Dex was associated with reduced POAF in studies with younger patients and smaller proportion of males, but not in studies with older patients or larger proportion of males. These findings indicated that perioperative administration of Dex may reduce the risk of POAF after cardiac surgery, particularly in Asians. Moreover, age and sex of the patients may affect the potential preventative efficacy of Dex on POAF.

Compared with previous meta-analyses of the same fields, our study has multiple strengths. Firstly, 15 up-to-date RCTs were included, which represents the most recent understanding of the influence of Dex on POAF after cardiac surgeries. In addition, strict inclusion criteria were applied, which avoided the confounding effects of including studies with overlapped patients. Finally, multiple sensitivity and subgroup analyses were performed to shown the stability of the results. Results of meta-analysis are consistent with the previous observed potential antiarrhythmic efficacy of Dex. An early study showed that the potential anti-arrhythmic efficacy of Dex could be related to its inhibition on sympatholytic properties and activation of the vagus nerve (36). A subsequent retrospective case series supported that Dex may have novel antiarrhythmic properties for the acute termination of reentrant supraventricular tachycardia (37). A recent experimental study in rat model of ischemic cardiomyopathy (ICM) showed that Dex conferred anti-arrhythmic effects in the context of ICM via upregulation of connexin 43 and suppression of inflammation and fibrosis (38). Future studies are needed to determine the exact pharmacological mechanisms that underlying the possible anti-arrhythmic efficacy of Dex.

Results of subgroup analyses showed that Dex reduced POAF after cardiac surgery in Asian studies, but not in non-Asian studies. Previous studies showed that Caucasians are associated with higher risk of POAF after cardiac surgery than patients of other ethnicity, including the Asians (39, 40). In our meta-analysis, the incidence of POAF in patients of control group from non-Asian countries was also higher than that from Asian countries (24.1 vs. 17.9%). These findings may suggest that Dex is more effective in reducing POAF after cardiac surgery in low-risk populations. Subgroup analyses also showed that Dex was associated with reduced POAF in studies with younger patients and smaller proportion of males, but not in studies with older patients or larger proportion of males, suggesting the potential preventative role of Dex on POAF may be more effective in young patient and in the females. The possible reasons for the findings are also to be determined. It has been shown that advanced age is a risk factor for POAF after cardiac surgery (41). Besides, men have been suggested to have higher incidence of POAF than women after CABG (42). Taken together, results of subgroup analysis may suggest that Dex was more effective for the prevention of POAF in low-risk patients. Since high-risk patients such as aged men are likely to have multiple comorbidities for POAF, and a single intervention of Dex may not be effective. These findings should be validated in large-scale RCTs.

Currently, Dexmedetomidine was recommended to be applied with a loading dose (0.4–1 μg/kg) and continuous infusion (0.2–0.8 μg/kg/h). Results of subgroup analysis showed no difference in reducing POAF after cardiac surgery between studies with and without a loading dose of Dex. Considering the possible adverse effect related with loading dose of Dex (such as hemodynamic instability and possible induction of severe arrhythmia) (43), continuous Dex infusion may be adequate for these patients. In addition, influence of Dex infusion timing on POAF is also clinically important. Although the subgroup difference was not significant, results of subgroup analysis indicated that Dex infusion in ICU after the surgery may be adequate for prevention of POAF (Table 4, 9 studies, OR = 0.64). Future large-scale RCTs are needed to determine whether post-operative use of Dex confers similar efficacy in preventing of POAF as compared to pre-operative use of Dex.

Our study has some limitations. First, the meta-analysis was based on data from study level rather than individual patient data. Accordingly, influences of a few key characteristics on the possible preventative role of Dex for POAF could not be analyzed in our meta-analysis, such as left atrial volume (44), renal function (45), and concurrent cardiovascular medications including perioperative use of beta-blockers, amiodarone, and electrolyte replacement protocol for K and Mg etc. (46), which have all been suggested to affect the risk of POAF after cardiac surgery. Moreover, the sample sizes of the included RCTs varied significantly, most of which were of small scale. Therefore, large-scale RCTs are still needed to validate the findings of the meta-analysis. In addition, weight is one of critical risks leading POAF after cardiac surgery (47). However, only five of the included studies reported body weight, while another five reported BMI. The limited available datasets and differences in body weight and BMI reported in the studies prevented further analyses in subgroup analyses. Besides, quality of some included studies is not good, which may affect the reliability of the findings. However, subgroup analysis did not support that difference in quality score may significantly affect the results. Finally, the optimal regimens of Dex administration for prevention POAF remains to be determined, such as dose, initiating time, and duration. Future studies are warranted in this regard.

In conclusion, results of this updated meta-analysis suggested that perioperative administration of Dex may reduce the risk of POAF after cardiac surgery, particularly in Asians. More studies are warranted to determine whether the age and sex of the patients may affect the potential preventative efficacy of Dex on POAF.

## Data Availability Statement

The original contributions presented in the study are included in the article/supplementary material, further inquiries can be directed to the corresponding author/s.

## Author Contributions

SP, GC, and PL contributed to the conception and design of the study. SP and HY performed literature search, study identification, quality evaluation, and data extraction. SP, JW, and HY performed the statistical analysis. SP, HY, GC, and PL wrote the first draft of the manuscript. All authors contributed to manuscript revision, and read and approved the submitted version.

## Funding

This study was supported by the Pudong New Area Commission of Science Technology of Shanghai (PKJ2018-Y17).

## Conflict of Interest

The authors declare that the research was conducted in the absence of any commercial or financial relationships that could be construed as a potential conflict of interest.

## Publisher's Note

All claims expressed in this article are solely those of the authors and do not necessarily represent those of their affiliated organizations, or those of the publisher, the editors and the reviewers. Any product that may be evaluated in this article, or claim that may be made by its manufacturer, is not guaranteed or endorsed by the publisher.
